# Imaging with Coherent X-rays: From the Early Synchrotron Tests to SYNAPSE

**DOI:** 10.3390/jimaging7080132

**Published:** 2021-08-04

**Authors:** Giorgio Margaritondo, Yeukuang Hwu

**Affiliations:** 1 Faculté des Sciences de Base, Ecole Polytechnique Fédérale de Lausanne (EPFL), CH-1015 Lausanne, Switzerland; 2 Institute of Physics, Academia Sinica, Taipei 11529, Taiwan; phhwu@sinica.edu.tw; 3 Department of Engineering Science, National Cheng Kung University, Tainan 70101, Taiwan; 4 Brain Research Center, National Tsing Hua University, Hsinchu 30013, Taiwan

**Keywords:** X-rays, synchrotron, coherence, phase contrast

## Abstract

The high longitudinal and lateral coherence of synchrotron X-rays sources radically transformed radiography. Before them, the image contrast was almost only based on absorption. Coherent synchrotron sources transformed radiography into a multi-faceted tool that can extract information also from “phase” effects. Here, we report a very simple description of the new techniques, presenting them to potential new users without requiring a sophisticated background in advanced physics. We then illustrate the impact of such techniques with a number of examples. Finally, we present the international collaboration SYNAPSE (Synchrotrons for Neuroscience—an Asia-Pacific Strategic Enterprise), which targets the use of phase-contrast radiography to map one full human brain in a few years.

## 1. Introduction: Radiography without and with Coherence

Radiography was invented by Wilhelm Roentgen just a few hours after his discovery of X-rays [1,2]. To produce images, he exploited only one of the interactions between the radiation and the objects: absorption. In reality, such interactions are more complicated [3,4,5,6,7]: wave phenomena such as refraction, interference, and diffraction complicate the interpretation of the images and may cause unwanted backgrounds. But they also enhance the information carried by the images and the opportunities to exploit it [3,4,5].

Such opportunities, however, remained largely unexploited for about one century. The detection of wave-like phenomena when electromagnetic radiation interacts with objects requires a special property called “coherence” [3,4,8]. We shall discover later the different aspects of coherence using simple arguments. And we shall specifically see why coherence is very limited for X-rays from conventional sources.

Starting in the 1960s, unconventional synchrotron radiation X-ray sources became available, which had high coherence levels [3,4]. Coherence-based radiography techniques became practical, impacting many research domains [5].

These facts notwithstanding, even today imaging with coherent X-rays is practiced only by a small portion of its potential user community. This limitation is partly caused by an insufficient understanding of the techniques, their foundations, and their effectiveness. Unfortunately, most descriptions of coherence-based radiography cannot be understood without a rather sophisticated physics background [8,9].

The primary objective of the present text is to fill this gap. We begin with a very simple description of coherence-based radiography, avoiding formalism and using elementary examples to introduce the essential notions and techniques. Then, we illustrate the practical use of such techniques with a few concrete cases. 

A discussion will follow, again in simple terms, of some advanced coherence-based imaging strategies. Finally, we illustrate the general potential impact of coherent radiography by presenting the recently inaugurated international project SYNAPSE [10], which targets in the forthcoming years the map of an entire human brain at the cellular level. 

## 2. Coherence-Based Imaging Mechanisms

A complete treatment of the image formation process in radiography would require a detailed theory of X-ray scattering by the electrons in the objects [3,4]. One would thus discover that both elastic (interference, diffraction, refraction) and inelastic (absorption, inelastic scattering) phenomena are present. And that elastic scattering is linked to the Fourier transform of the electronic charge distribution.

One could thus imagine the retrieval of the charge distribution from the images by inverse Fourier transforms, and from it, the derivation of the structural characteristics of the objects. However, the practice of radiography is very different from this idealized view, for both fundamental and practical reasons. 

In essence, the vast majority of radiography applications still rely only on absorption and on the visual inspection of the corresponding images. As mentioned, coherence-based imaging is used only, in a rather limited way, by a small community of specialists. 

When trying to export it beyond this restricted group, one cannot expect the audience to accept complex presentations. The objective of most clinical radiologists is just to “see” things in the objects, with as little processing and interpretation as possible. In their eyes, coherence-based techniques are justified only if they facilitate the observation of interesting features.

**Figure 1:**
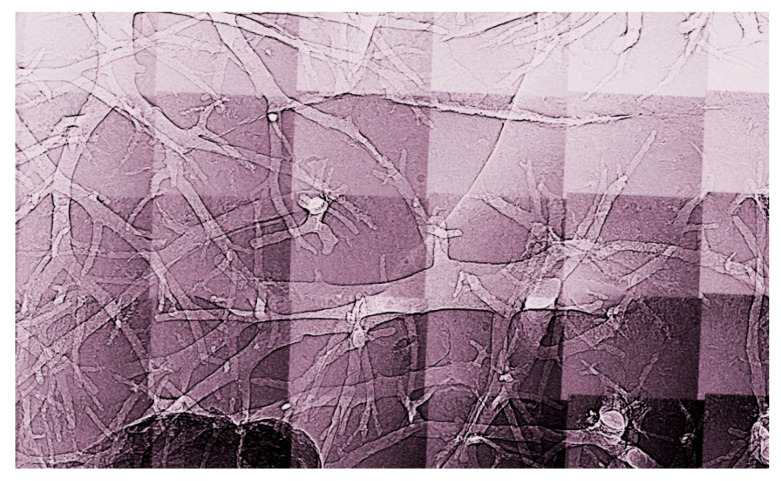
An example of a coherence-based radiograph: the enhanced edges of microscopic vessels in a mouse liver [13]. Horizontal image size: 4.2 mm.

**Figure 2:**
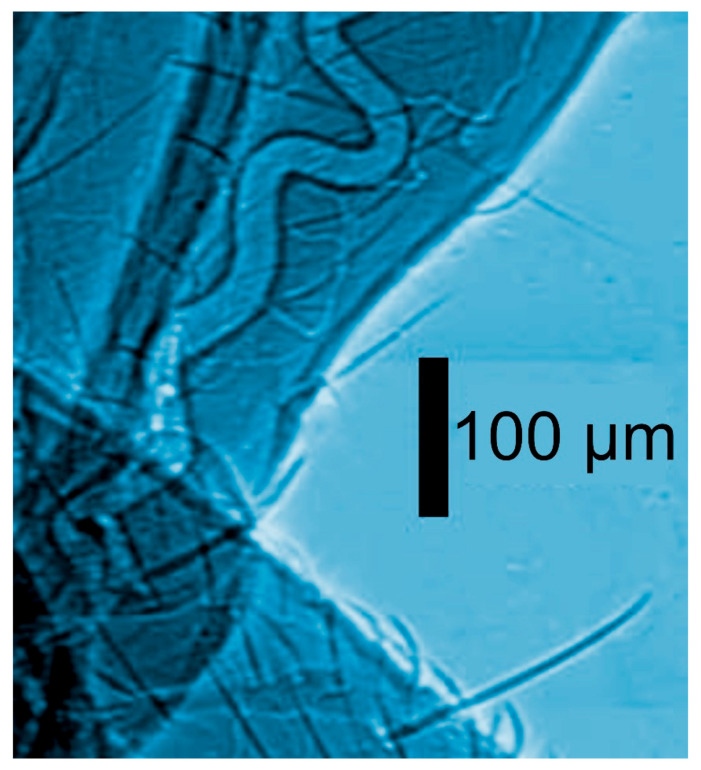
Coherence-based radiograph of part of an insect [12].

**Figure 3:**
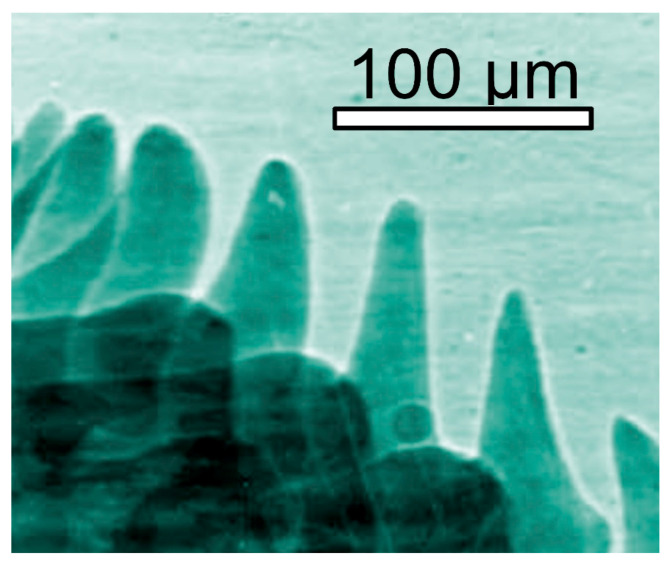
Coherence-based radiography can be also applied to live objects, such as small fish [12].

**Figure 4:**
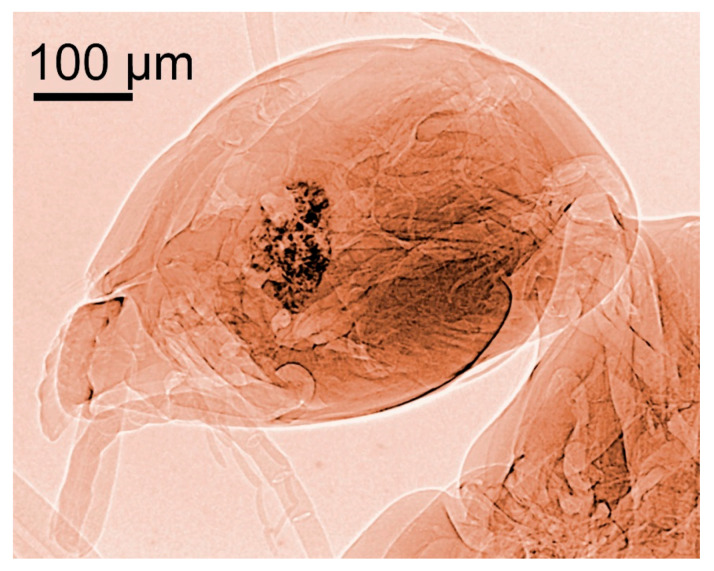
Coherence-based radiograph of the head of a housefly [13].

**Figure 5:**
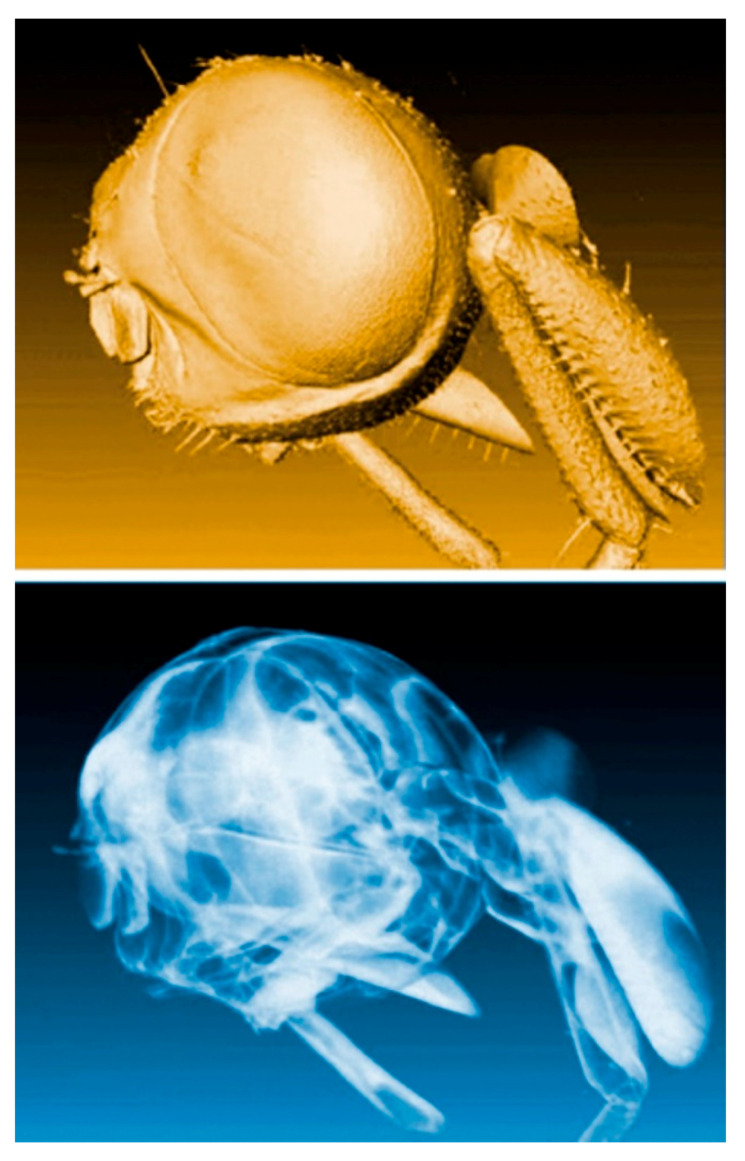
Two other coherence-based X-ray images of part of a housefly [14].

And this is often true, as shown by the examples of Figure 1, Figure 2, Figure 3, Figure 4 and Figure 5 [11,12,13,14], which present a series of images taken with coherent X-rays from synchrotron sources. We note in these radiographs a common and very important feature: coherence enhances the image quality by boosting the visibility of edges between different regions of the object (and between the object and vacuum). This makes the pictures more useful without complex data processing.

**Figure 6:**
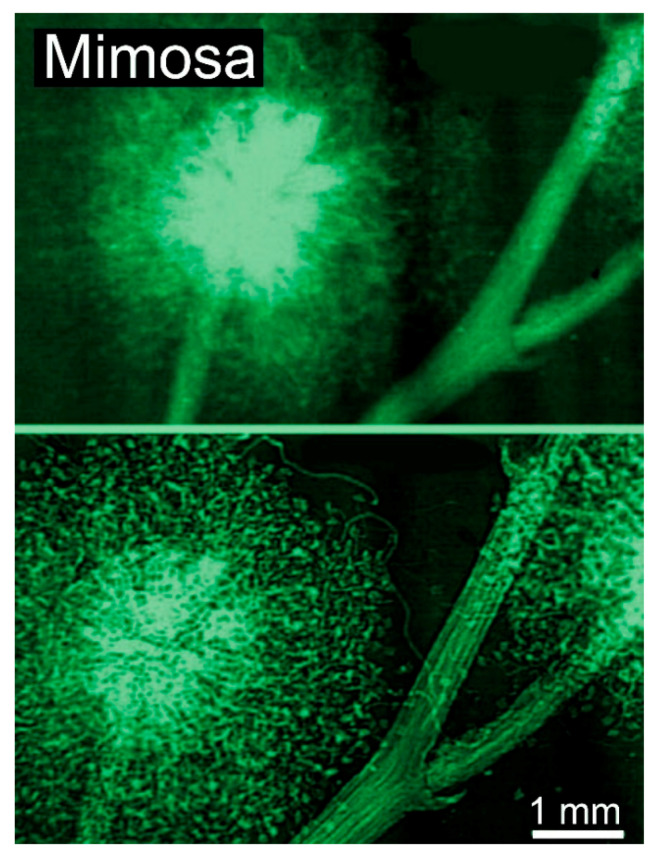
Direct comparison of a conventional absorption radiograph and a coherence-based radiograph: mimosa flower [12]. The result was obtained on the Elettra synchrotron in Trieste under the leadership of Giuliana Tromba.

Therefore, our first objective here is to understand how coherent X-rays produce the edge enhancement. The impact of this enhancement is vividly illustrated by Figure 6, which compares two radiographs of the same object without and with coherence-based contrast [12]. In the first case, only absorption is exploited, obtaining an image of intermediate quality. In the second case, the radiograph has sharper features and strong edges, delivering much more information.

The phenomena that improve the second image of Figure 6 are related to the wave nature of X-rays and specifically to their “phase”, a notion intimately related to coherence. Hence, the corresponding radiography techniques are collectively called “phase-contrast imaging”. 

To introduce the idea of “phase”, we shall consider the following, the simple mathematical form of an electromagnetic plane-wave. This is a sinusoidal electric field of amplitude *E*_o_, propagating with the time *t* in the *x*-direction:

*E*(*x*,*t*) = *E*_o_sin[(2*π/λ*)(*x* − *vt*)], (1)

where *λ* is the wavelength and *v* is the velocity. 

The “phase” of the wave is the argument (2*π/λ*)(*x* − *vt*) of the sine function in Equation (1). To verify that this function does propagate with time, note that *E*(*x*,*t*) is the same for combinations of *x* and *t* that give the same phase. Consider for example the phase for *x* = zero, *t* = zero, which is zero. And the phase for *x* = Δ*x*, *t* = Δ*t*, which remains equal to zero if Δ*x* − *v*Δ*t* = zero, i.e., if *v* = Δ*x*/Δ*t*. This shows that the wave of Equation (1) propagates indeed with speed *v*. 

X-ray absorption in a material decreases the amplitude *E*_o_ but does not affect the phase. Thus, absorption-based radiographs do not exploit phase-related phenomena. With coherent X-rays, such phenomena can contribute to the images, improving them and increasing their information content [11,12,13,14], as seen in Figure 1, Figure 2, Figure 3, Figure 4, Figure 5 and Figure 6. 

**Figure 7:**
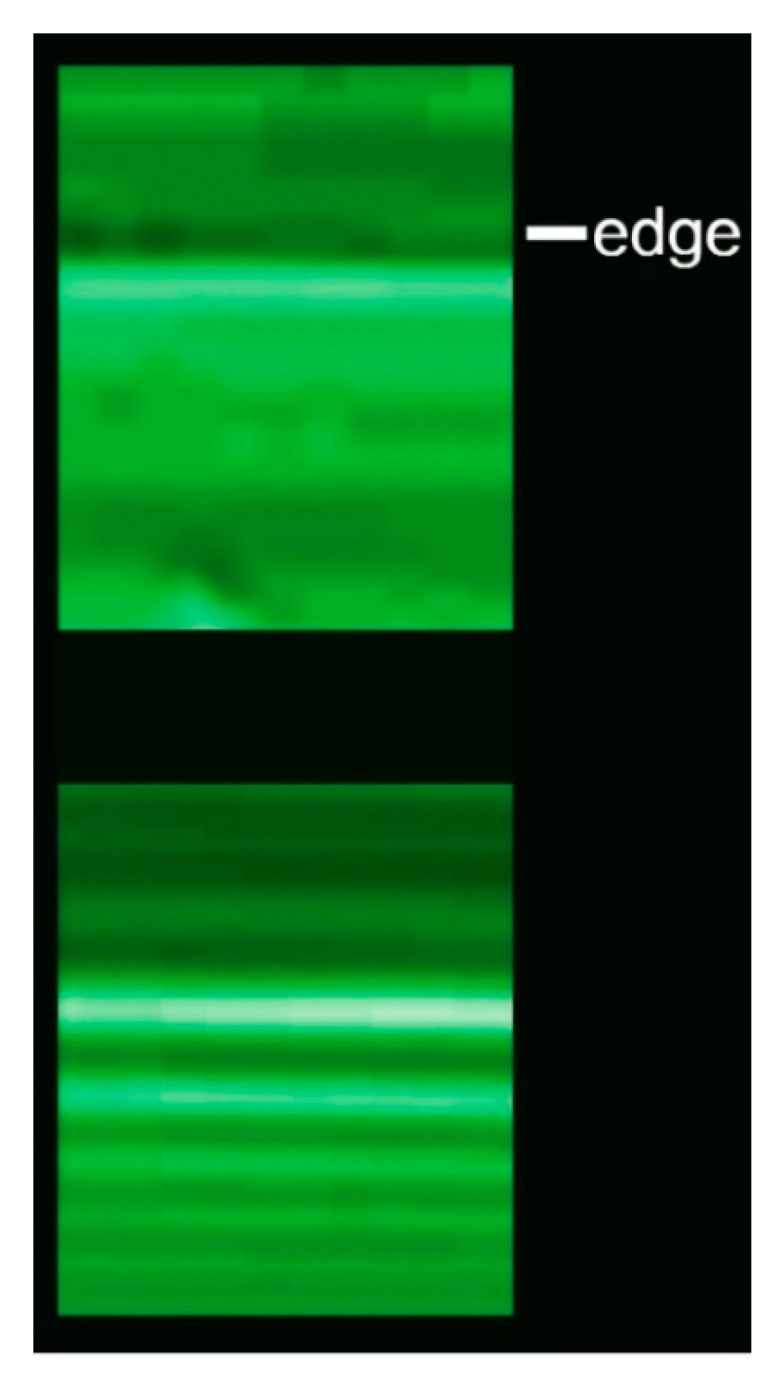
Two radiographs of the edge of an optics fiber, taken with coherent X-rays for two different object-detector distances, *r*_o =_ 0.25 m (top image) and *r*_o =_ 1.4 m [15,16]. Edge enhancement due to refraction dominates in the first case, whereas Fresnel edge diffraction accounts for the fringe sequence in the second.

Before starting our analysis of phase-related effects, we must discover one important point using Figure 7. Here, the edge between an optics fiber and vacuum is imaged with coherent X-rays.

The radiographs were taken using two different distances *r*_o_ between the fiber and the X-ray detector. We can see two different types of edge enhancement: in the top radiograph, the edge corresponds to a pair of dark-bright fringes. In the bottom one, there is instead a sequence of fringes. 

This indicates that at least two different mechanisms cause edge enhancement. Our immediate objective is to understand both of them as well as their interplay.

### 2.1. Edge Enhancement by Refraction

**Figure 8:**
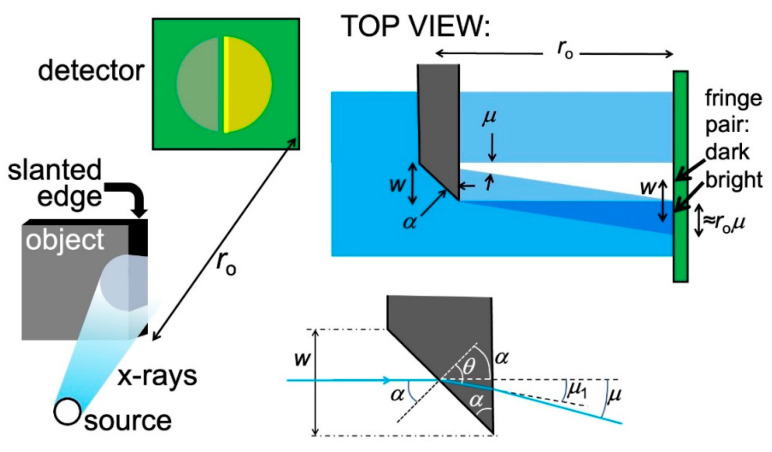
Left: refraction of X-rays passing through a slanted edge between an object and vacuum cause a characteristic pair of bright-fark fringes. A similar phenomenon occurs for a slanted edge separating two different object regions. Top right: analysis of the fringe geometry. Bottom right: enlarged scheme of the edge showing the parameters used ion the analysis.

The first one of the two mechanisms is related to refraction [15,16] and produces fringe pairs like those of the top image of Figure 7. This is the mechanism active in most phase-contrast radiographs, e.g., those of Figure 1, Figure 2, Figure 3, Figure 4 and Figure 5. We can understand it using the idealized object of Figure 8: a partially absorbing plate separated from the vacuum by a slanted edge with an angle *α*. A similar analysis would also apply to a slanted edge between two different object areas [16].

The portion of the X-ray beam that passes through the slanted edge is subject to refraction and deviated by an angle *μ*. This produces on the detector (Figure 8, top right) a zone with enhanced illumination adjacent to a dark one: a pair of bright-dark fringes.

Let us discover the practical conditions for observing such fringes. On the detector, the distance between the two fringes equals the edge width *w*. And, more in general, the inter-fringe distance depends on the specific shape of each edge. So, we cannot formulate a general rule for seeing the refraction edge enhancement, valid for all edges in all objects. But we can reach some useful empirical conclusions.

First, the visibility of a dark-bright fringe pair depends on the object-detector distance *r*_o_ and the deviation angle *μ*. In fact, the width of each fringe on the detector is ≈*r*_o_*μ*. If *r*_o_ is small, the fringes could be too narrow for the lateral resolution of the detector to reveal them. By increasing *r*_o_, one can broaden the fringes and start detecting them, compensating for the limited resolution.

If *r*_o_ is further augmented, the fringe width ≈ *r*_o_*μ* keeps increasing whereas the fringe distance *w* does not change. Optimum visibility of the fringe pair is reached when *r*_o_*μ* ≈ *w*. This condition depends on the edge shape: one cannot identify a distance *r*_o_ that optimizes all edges. However, *r*_o_ can be empirically adjusted to obtain the best enhancement for the majority of the edges in the object under investigation, or at least for the most interesting ones.

**Figure 9:**
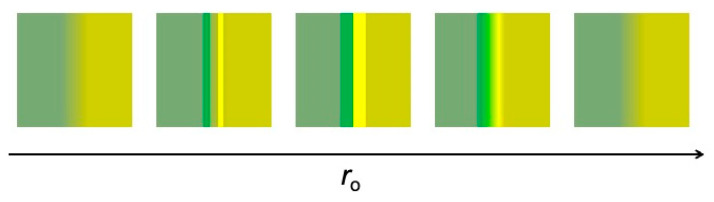
The visibility of a refraction fringe pair changes in a non-monotonic way with the object-detector distance, *r*_o_.

If *r*_o_ is increased beyond optimum visibility, the fringe widths become larger than the inter-fringe distance. The pair becomes difficult to detect and eventually fades away. Therefore, the overall evolution with *r*_o_ of the fringe pair visibility is not monotonic, as summarized in Figure 9.

What makes the refraction edge fringe pairs detectable in practice, as seen in Figure 1, Figure 2, Figure 3, Figure 4, Figure 5 and Figure 6? Consider that the X-ray beam deviation angle *μ* depends on the refractive index *n* according to Snell’s refraction law. For X-rays, *n* is very close to 1 and—contrary to visible light—smaller than 1. Thus, it can be written as:

*n* = 1 − *δ*, (2)

where *δ* is very small: typical values for X-ray photons of energy 1–10 keV (with a wavelength *λ* of the order of 1–10 angstrom) are in the 10^−4^–10^−5^ range or even smaller [17].

A simple but tedious trigonometric calculation (Appendix A) shows that:

*μ* ≈ (tg*α*)*δ*, (3)

where the coefficient tg*α* depends on the edge geometry and is typically not far from 1. 

So, the above values of *δ* correspond to deviation angles in the 10^−4^–10^−5^ radians range: for a detector-object distance *r*_o_ ≈ 1–10 m, they would give refraction fringe widths of the order of 10–1000 µm. 

This is in agreement with the empirical findings and with the typical detector spatial resolution, explaining why refraction edge enhancement can be observed. Note that 10–1000 µm is also the typical width of the edges for which refraction enhancement can be seen.

### 2.2. Coherence in Refraction Edge Enhancement

To start understanding X-ray coherence and its role in edge enhancement, let us consider the wavelengths of the X-ray beam in Figure 8. So far, we implicitly assumed that this beam is “monochromatic”, i.e., it includes only one wavelength, *λ*. More realistically, however, the beam consists of a band of wavelengths of width δ*λ*. In principle, this could decrease or even wipe out the edge enhancement caused by refraction.

This is expressed by saying that the bandwidth δ*λ* defines the X-ray beam coherence. More specifically, it determines what is called the “longitudinal coherence”.

**Figure 10:**
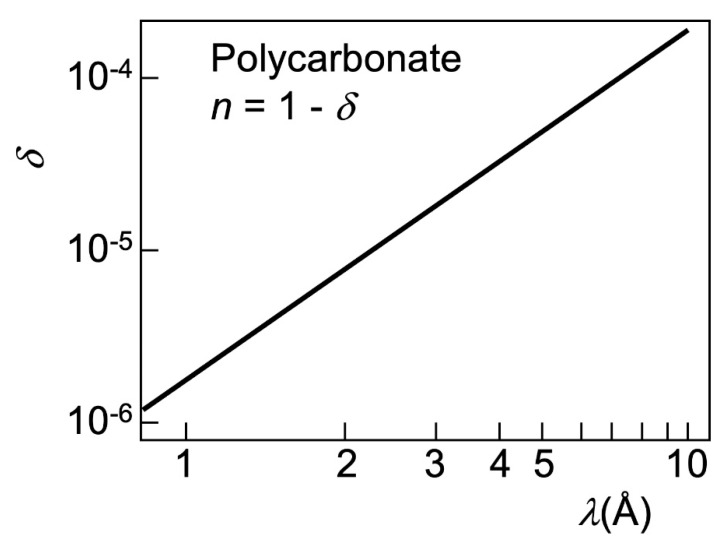
Refractive index of polycarbonate as a function of the wavelength in the X-ray range. These results are representative of the general behavior and magnitudes for different materials. Data from [17].

To understand the relation between the “longitudinal coherence” parameter δ*λ* and the edge enhancement, consider that the wavelengths do influence refraction, since *δ* in Equations (2) and (3) changes with them (see Figure 10). For example, a small *δ* makes (Equation (3)) the deviations *μ* negligible and may impede the refraction edge enhancement. 

Consider a typical synchrotron X-ray beam for imaging, centered at *λ* ≈ 1 Å and with a bandwidth δ*λ* = 1 Å, extending from 0.5 to 1.5 Å. We see in Figure 10 [17] that the corresponding values of *δ* roughly range from 10^−6^ to 10^−5^. Suppose that refraction enhancement applies to an edge of width *w* = 10 µm with a slanting angle *α* = π/4. The optimized distance *r*_o_ for the central wavelength *λ* ≈ 1 Å—roughly corresponding to *δ* ≈ 2 × 10^−6^ and (Equation (3)) to *μ* ≈ 2 × 10^−6^ radians—is *r*_o_ ≈ *w*/*μ* ≈ 5 m. 

Consider now, for this optimized *r*_o_, the other wavelengths in the band 0.5–1.5 Å and their *δ*-values. Short wavelengths, 0.5–1 Å, correspond to narrower fringes than for the central wavelength, but to the same fringe positions. If anything, this increases the fringe visibility. 

Long wavelengths in the band, 1–1.5 Å, correspond instead to broader fringes with less visibility. However, such wavelengths are more strongly absorbed by the material and contribute less to the fringes. 

These intuitive arguments, corroborated by abundant empirical evidence, reveal a fundamental fact [16]: the finite X-ray wavelength bandwidth has a limited impact on edge enhancement by refraction. More specifically, the natural bandwidth of all synchrotron radiation sources is by itself adequate to detect this type of enhancement. This is even more true for undulator sources than for bending magnets or wigglers, since their bandwidth is automatically narrower [3,4] facilitating the refraction edge enhancement. 

Therefore, one does not need to decrease δ*λ* by filtering synchrotron radiation with additional devices, the so-called “monochromators”. This is very good, since monochromators sharply decrease the X-ray flux. Avoiding them strongly increases the signal in the imaging experiment. Historically, this played a crucial role in the expansion of phase-contrast radiography [16]. 

These facts can be summarized by saying that synchrotron radiation possesses sufficient “longitudinal coherence” for refraction edge enhancement. However, this is only one of the two aspects of coherence. The second one is “lateral coherence”, which is related to the geometry of the X-ray source. 

To introduce the notion of “lateral coherence”, note that an excessively large source size in Figure 8 could “blur” the refraction-induced deviations and could jeopardize edge enhancement by refraction. This is why this type of enhancement could be observed only when small-size synchrotron sources came into play. For the present synchrotron facilities, “lateral coherence”—and in particular the source size—is largely sufficient for edge enhancement by refraction [3,4,5].

We shall further elaborate on “lateral coherence” later. But we can immediately realize that for phase-contrast imaging the X-rays must possess sufficient levels of both kinds of coherence, longitudinal and lateral. And this is true for the synchrotron sources of today without requiring further measures like monochromatization. 

### 2.3. Fresnel Edge Diffraction

**Figure 11:**
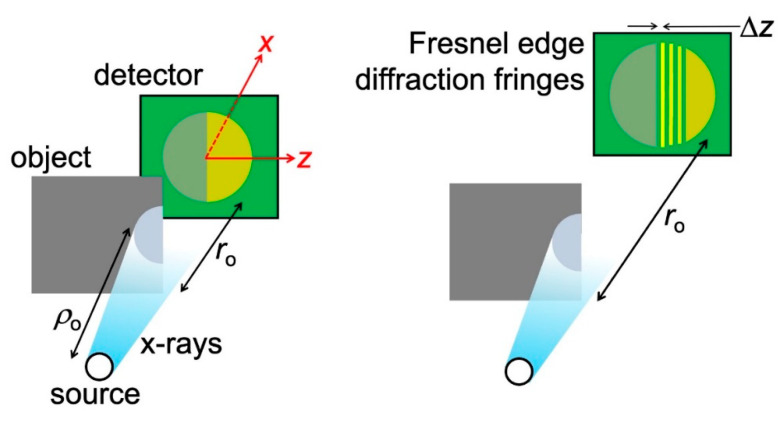
X-rays intercepting a straight edge between an object and vacuum can cause the “Fresnel edge diffraction” fringes [15,16]. A similar phenomenon occurs for the straight edge between two different regions of the object.

Let us now go back to Figure 7, specifically to its bottom image: a sequence of fringes rather than a bright-dark fringe pair. This sequence cannot be explained by refraction at a slanted edge. To find its cause, consider the simple case of Figure 11: an object similar to that of Figure 8 but with a straight edge [15]. 

If absorption was the only active mechanism, then the detected image would simply consist of two regions with different intensities, as seen on the left-hand side of Figure 11. However, when the object detector-distance *r*_o_ is large enough (right-hand side of the figure), one also sees a fringe sequence like that in the bottom part of Figure 7. The cause is the phenomenon known as “Fresnel edge diffraction” [15], which occurs both for visible light and for X-rays, at edges between the object and vacuum or between different parts of the object.

Elementary optics shows [15] that the distance between the first bright fringe and the first dark one in the sequence is:

Δ*z* = (*λr*_o_)^1/2^. (4)

Specifically, Equation (4) is valid for the realistic case *r*_o_ << *ρ*_o_, where *ρ*_o_ is the distance between the X-ray source and the object. By deriving it, we can discover the origin of Fresnel edge diffraction.

Consider, in fact, a ray that reaches the detector after having been scattered by the edge. On the detector, its wave is combined with that of the rays that reach the same point without being scattered. Constructive interference, corresponding to bright fringes, occurs when the path difference equals an integer number of wavelengths *λ*. And dark fringes occur when the path difference equals an odd-integer multiple of *λ*/2. 

The object-detector path for rays without scattering is *r*_o_. For a ray scattered by the edge and arriving at the position *z* on the detector, the path is (*r*_o_^2^ + *z*^2^)^1/2^.

The path difference for the first bright fringe is zero. And that for the adjacent dark fringe, occurring at *z* = Δ*z*, is *λ*/2. Thus:

[*r*_o_^2^ + (Δ*z*)^2^]^1/2^ − *r*_o_ = *λ*/2; *r*_o_^2^ + (Δ*z*)^2^ = (*λ*/2 + *r*_o_)^2^, *r*_o_^2^ + (Δ*z*)^2^ = *λ*^2^/4 + *λr*_o_ + *r*_o_^2^, (Δ*z*)^2^ = *λ*^2^/4 + *λr*_o_, (5a)

which, since for short-wavelength X-rays *λ* << *r*_o_, gives:

(Δ*z*)^2^ ≈ *λr*_o_, (5b)

indeed, Equation (4).

The practical detection of a Fresnel diffraction fringe sequence is possible in practice only under certain conditions. These include sufficient spatial resolution and high coherence. 

Specifically, the spatial resolution of the detector must be sufficient to distinguish adjacent fringes, i.e., it must be better than Δ*z*. From Equation (4), we see that Δ*z* increases with *r*_o_. Thus, one can again compensate for the limited spatial resolution of the detector by placing it farther away from the object.

Concerning “longitudinal coherence”, we must consider the presence of *λ* in Equation (4). Assume once again that the X-ray beam is not “monochromatic” but has a bandwidth δ*λ*. This “blurs” the inter-fringe distance from Δ*z* to δΔ*z* and could make the fringes impossible to detect. 

The fringe sequence is still visible if the “blurring” δΔ*z* does not exceed the inter-fringe distance itself: δΔ*z* < Δ*z*. From Equation (4) we get:

ln(Δ*z*) ≈ [ln(*r*_o_) + ln(*λ*)]/2, (6a)

and, taking the derivative:

δΔ*z*/Δ*z* ≈ δ*λ*/(2*λ*); (6b)

Thus, the requirement δΔ*z* < Δ*z* is satisfied if:

δ*λ*/*λ* < 2 (7)

Equation (7) is the condition for “longitudinal coherence” in this particular case.

In addition, there is also a source geometry condition for “lateral coherence”. So far, our analysis implicitly assumed an X-ray source with a small size. What happens if the source has a large transverse dimension, for example in the *z*-direction? 

In essence, the emission of each source point produces an individual diffraction pattern. The superposition of all patterns can wash out the fringes, making it impossible to detect them. Hence, we find here again that “lateral coherence” requires a small source.

### 2.4. Refraction-Diffraction Interplay

For a slanted edge, the fringe pair caused by refraction and the fringe sequence caused by diffraction can be combined. Both become visible if *r*_o_ is large enough to compensate for the limited spatial resolution of the detector. There is, however, an important difference between the two kinds of fringes. 

The visibility of a diffraction fringe sequence continues to improve as *r*_o_ is increased. On the contrary, we see in Figure 9 that the refraction fringe pair reaches optimum visibility and then fades away. This determines the interplay between the two mechanisms and explains, for example, the results of Figure 7: refraction fringes dominate for small values of *r*_o_ and diffraction fringes for large values [16]. 

In the practice of coherence-based radiography, refraction fringe pairs are most frequently observed whereas diffraction fringe sequences are seldom seen. This is due to the fact that complicated edge shapes yield complex diffraction sequences, difficult to observe. Whereas refraction fringe pairs are typically simple and intense, and therefore easy to see.

These empirical facts can be exploited to optimize the image quality by tuning *r*_o_. The best approach is to boost the visibility of refraction fringe pairs, which are most effective in producing edge enhancement. Note again, however, that this optimization is not universal and must be repeated for each object.

**Figure 12:**
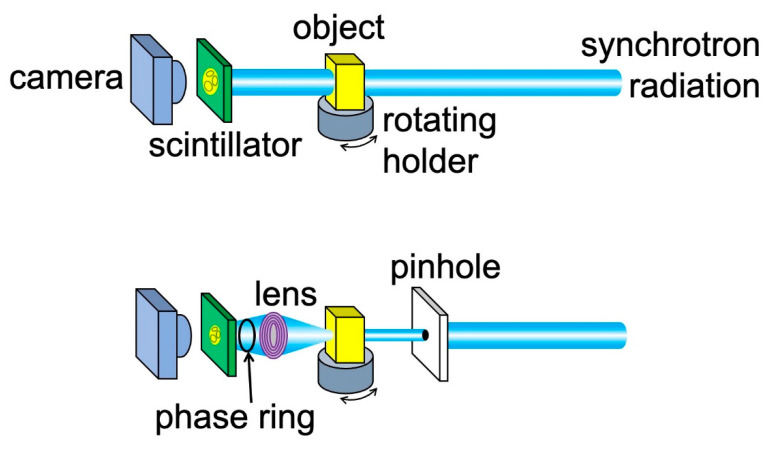
Two online experimental geometries to implement a radiography system based on edge enhancement. (**Top**): the simplest arrangement, which does not include any magnification. (**Bottom**): geometry for radiographic microscopy, based on an X-ray lens (see Section 2.5). This configuration also offers the option of a phase-adjusting device to enhance the contrast, as mentioned later in the text.

The practical way to implement edge-enhanced radiography is by using the geometry of Figure 8 and Figure 11, which is explicitly illustrated by the top part of Figure 12: the object and the detector are placed online along the X-ray beam direction. A typical detector includes a scintillator that converts X-rays into visible light, plus a device (e.g., a camera) to acquire the resulting visible images. The X-ray beam can be filtered by a monochromator, although we have seen that in many cases edge-enhanced radiography does not require it. The beam geometry can be improved, for example making it more parallel, by a suitable condenser.

### 2.5. Microscopy

The simple online geometry of the top part of Figure 12 does not fully exploit all the performances of coherence-based radiography, in particular its potentially high spatial resolution. An alternate strategy, shown in the bottom part of the same figure, is to place an X-ray lens between the object and the scintillator, magnifying the image and boosting its resolution. The result is a powerful X-ray microscope.

The technical problem with this approach is finding suitable lenses for X-rays. The devices for visible light, such as focusing mirrors or refractive lenses, cannot be easily transferred to the X-ray wavelength range. We have seen that X-ray refraction is very weak, since the index of refraction *n* is very close to 1 [17]. And the reflection of X-rays is also very limited, except at extreme glancing incidence.

**Figure 13:**
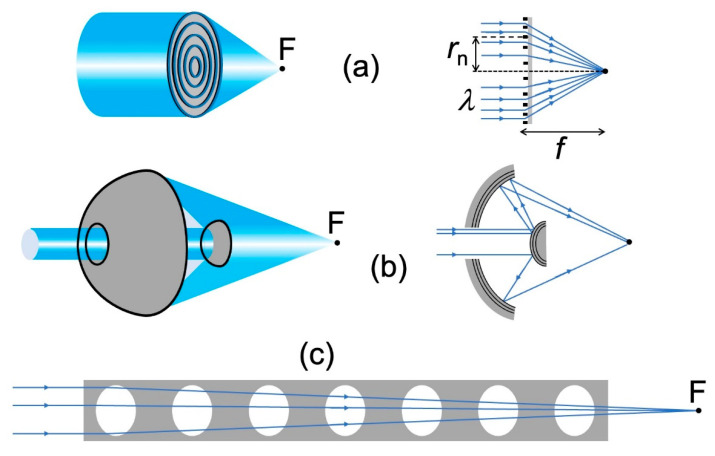
Three types of X-ray lenses: (**a**) Fresnel zone plate [12,14,18,19,20,21,22,23,24]; (**b**) Schwarzschild objective [25] with multilayer coatings to enhance the X-ray reflection at the relevant surfaces; (**c**) compound refractive lens [26].

As a consequence, special focusing devices must be invented and realized for X-rays: this is a crucial problem in the evolution of advanced radiography towards high spatial resolution. Major progress was made in recent years [12,14,18,19,20,21,22,23,24,25,26], and Figure 13 schematically shows three of the main technical solutions.

The two top pictures (Figure 13a) specifically illustrate the “Fresnel zone plates” (FZPs) [3,5,18,19,20,21,22,23,24] for X-rays. They are the counterparts of the common FZPs for visible light. And consist of alternating absorbing and transmitting, concentric circular zones. 

Constructive interference at the focal point F occurs if the difference in the path between the rays passing through the center and the *i*-th zone equals *i* full wavelengths:

(*r*_i_^2^ + *f*^2^)^1/2^ − *f* = *iλ*; (8a)

(*r*_i_^2^ + *f*^2^)^1/2^ = *iλ* + *f*; (8b)

*r*_i_^2^ = (*iλ* + *f* )^2^ − *f*^2^ = *λ*(*i*^2^*λ* + *if*), (8c)

which for short-wavelength X-rays becomes:

*r*_i_ ≈ (*λif* )^1/2^. (8d)

The presence of *λ* in Equation (8) implies that the typical size of X-ray FZPs is much smaller than for visible-light FZPs. Fabricating such minuscule devices is not easy [18,19,20,21,22,23,24], also because their size must be large enough to capture most of the X-ray beam emitted by the source rather than wasting it. This, according to Equation (8), requires a total number of zones, i.e., a maximum value *i*_MAX_ of *i*, as high as possible. 

The same equation gives for the width Δ_MAX_ of the outermost zone:

Δ_MAX_ ≈ {(*λi*_MAX_*f*)^1/2^ − [*λ*(*i*_MAX_ − 1)*f*]^1/2^} = (*λi*_MAX_*f*)^1/2^[1 − (1 − 1/*i*_MAX_)^1/2^] ≈ (*λi*_MAX_*f*)^1/2^{1 − [1 − 1/(2*i*_MAX_)]} = (*λi*_MAX_*f*)^1/2^[1/(2*i*_MAX_)] = (*λf*/*i*_MAX_)^1/2^/2. (9)

Equation (8) shows that the FZP radius is *r*_MAX_ ≈ (*λi*_MAX_*f*)^1/2^, which gives *i*_MAX_ ≈ *r*_MAX_^2^/(*λf*), so Equation (9) implies:

Δ_MAX_ ≈ *λf*/(2*r*_MAX_). (10)

This is a key result: a large FZP radius *r*_MAX_ requires a very narrow outermost zone width Δ_MAX_, which is very difficult to fabricate. This difficulty is augmented by the weak X-ray absorption of the blocking zones, which requires them to have a large thickness. Therefore, the outermost zones must be radially narrow but very thick, i.e., with a high “aspect ratio” [18,19,20,21,22,23,24].

**Figure 14:**
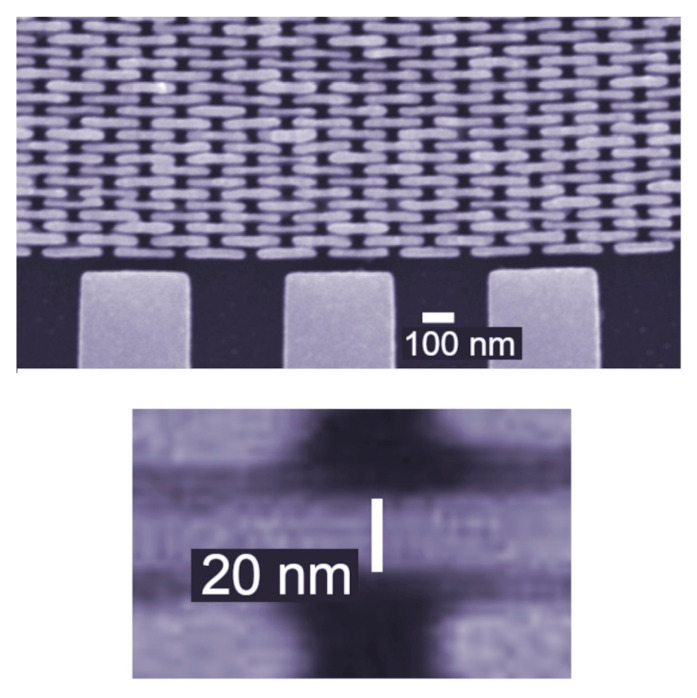
Micrograph showing the outermost zones of an X-ray FZP, with a zone width as small as 20 nm [18,19,20,21,22,23,24]. Also shown is the external support structure.

**Figure 15:**
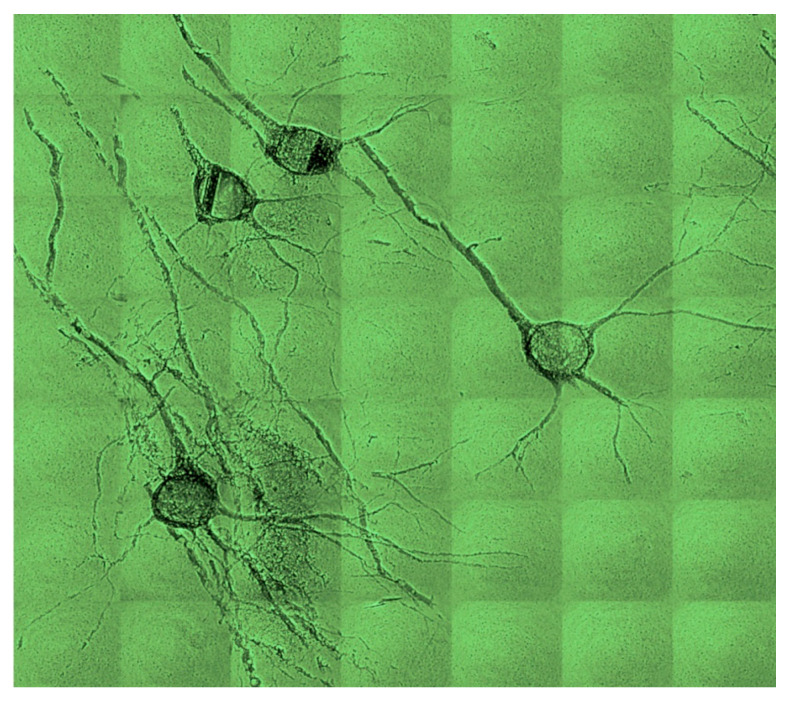
Test of magnification in the X-ray range by a Fresnel zone plate, which demonstrated a lateral resolution sufficient for the analysis of brain specimens at the cellular level [18,19,20,21,22,23,24]. Horizontal image size: 130 µm.

The mechanical fragility of such features makes fabricating FZPs for X-rays a very challenging task. This was successfully implemented, as shown in Figure 14: the outermost zone here is 20 nm thick and with a high aspect ratio. The results of Figure 15 [18] illustrate the fact that the X-ray FZPs now yield an X-ray micrograph with resolutions reaching the nanometer range.

Figure 13b shows another successful X-ray lens, the “Schwarzshild objective” developed under the leadership of the late Franco Cerrina [25]. The device is a combination of two mirrors, one convex and one concave.

One might be surprised that this lens works for X-rays, since it uses two reflections at near-normal incidence, which should be very weak at short wavelengths. The secret is the use of multilayer coatings that increase X-ray reflectivity. The feasibility of this approach was demonstrated by successful tests, as reported in Ref. [25].

Finally, Figure 13c shows a “compound refractive lens” (CRL) [26], consisting of a regular series of cavities along the axis of a material like beryllium. Since the refractive index of X-rays is below 1, refraction at the surfaces of each cavity produces focusing. Individually, one cavity would not be very effective. But the combination of several cavities in series produces satisfactory magnification, as reported for example in Ref. [26].

The combination of focusing/magnification with coherence-based imaging can be also improved by adding a phase-controlling element to an FZP microscope, as shown in the bottom part of Figure 12. The strategy was successfully tested [22] using a “Zernike ring” for phase adjustments. This allowed enhancing the phase-contrast while preserving the magnification. 

## 3. More about Coherence

Our previous discussion was focused on edge enhancement by refraction, which as mentioned dominates most applications of phase-contrast radiography. But other interesting coherence-based techniques exist that deserve at least a first look, as we shall discover in Section 5. For those, it is necessary to acquire a knowledge of coherence a bit more advanced than what we have learned so far.

**Figure 16:**
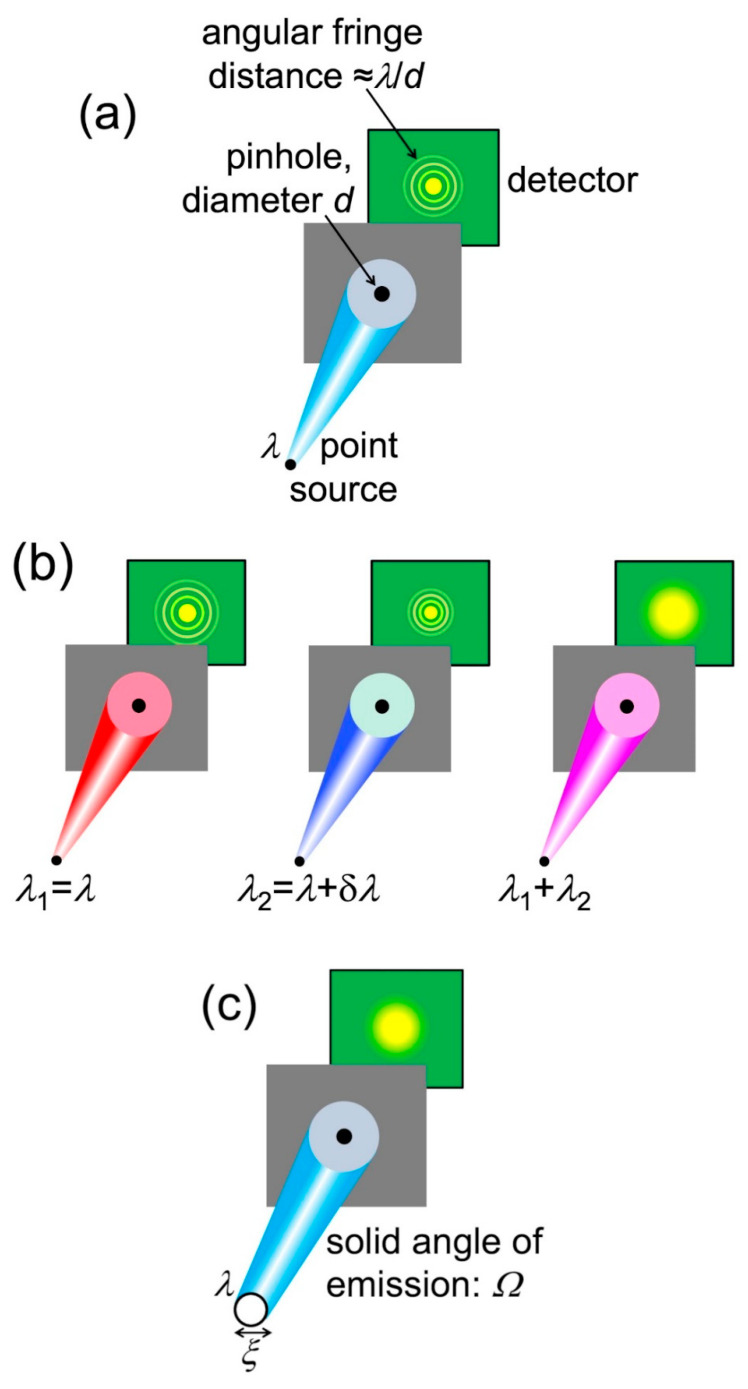
Description of coherence using pinhole diffraction. (**a**) If the source is point-like and only emits one wavelength, a diffraction pattern is always visible; (**b**) if the source emits different wavelengths (in this case two, separated by δ*λ*); the superposition of the corresponding patterns may wash out the fringes; (**c**) Likewise, an extended source could make the pattern fringes undetectable.

Let us start with a general empirical definition of coherence: “the property that makes it possible to detect wave-like phenomena”. To analyze its essential aspects, we can use anyone of such phenomena. Here, we shall select the simple case of diffraction by a narrow circular pinhole (Figure 16). This is an approximate simulation of diffraction phenomena caused by small local features of the object.

Pinhole diffraction produces a detectable pattern of circular bright-dark fringes only if the radiation has a sufficient level of coherence. What are the corresponding conditions? Let us start with a point-like source that emits only one wavelength *λ*, as shown in Figure 16a. A source of this kind always produces a visible pinhole diffraction pattern, thus it is fully coherent.

Let us now relax these conditions, assuming that the source is not “monochromatic” but emits a wavelength bandwidth δ*λ*, as in the right-hand image of Figure 16b. Elementary optics shows that the distance between two adjacent bright fringes in the pattern of Figure 16a is ≈*r*_o_*λ*/*d*, where *d* is the pinhole diameter.

The bandwidth δ*λ* “spreads” this inter-fringe distance to *r*_o_δ*λ*/*d*, blurring the fringe pattern. Which ceases to be visible when the spread becomes comparable to the distance between two adjacent fringes.

Thus, the requirement for the pattern to be observable is *r*_o_δ*λ*/*d* < *r*_o_*λ*/*d*, or:

δ*λ*/*λ* < 1. (11)

This is in this case the condition for “longitudinal coherence”, qualitatively equivalent to that of Equation (7). 

Note that this condition is not very stringent. As a curiosity, applied to sunlight it explains why we can observe wave-like phenomena in everyday life, such as the beautiful colored fringes of soap bubbles. Indeed, the detection bandpass of our eyes guarantees that Equation (11) is satisfied for the solar spectrum that we can see. 

### Lateral Coherence

In addition to “longitudinal coherence”, there is a “lateral coherence” (source geometry) condition for seeing the pinhole diffraction fringes. We assumed so far a point-like source, but Figure 16c shows a more realistic case: a finite circular source with diameter *ξ* and with an angular beam divergence corresponding to a solid angle *Ω*.

**Figure 17:**
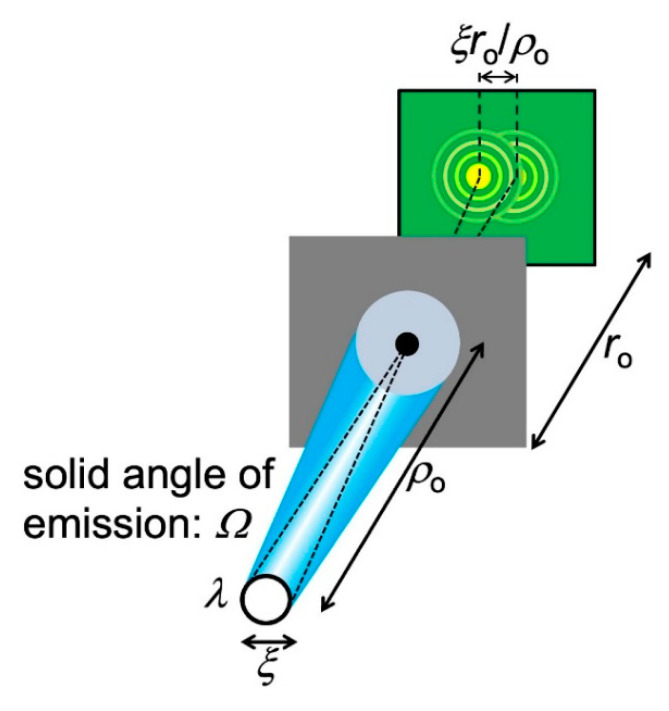
Analysis of the coherence conditions to observe a pinhole diffraction pattern when the source has an extended area.

The angular divergence implies that only a portion of the source emission passes through the pinhole and participates in diffraction. Look at Figure 17: calling again *ρ*_o_ the distance between the source and the object, the solid angle corresponding to the pinhole area *πd*^2^/4 is ≈ (*πd*^2^/4)/*ρ*_o_^2^. Therefore, the portion *η* of the emitted power that contributes to diffraction is:

*η* = [(*πd*^2^/4)/*ρ*_o_^2^]/*Ω*. (12)

As the divergence *Ω* decreases, *η* increases: a larger portion of the source emission contributes to the diffraction pattern, improving its signal-to-noise ratio. Conversely, if *η* decreases more of the emission is wasted as far as pinhole diffraction is concerned. 

Let us now bring into the picture (Figure 17) the finite size *ξ* of the source. Each source point acts as an emitter and produces a diffraction pattern centered at a specific position on the detector. The overall pattern is the superposition of all these individual patterns. And it could be no pattern at all, i.e., fringes may no longer be visible. 

To analyze this point, note in Figure 17 that the maximum distance between the centers of individual patterns is, on the detector, *ξ*(*r*_o_/*ρ*_o_). Fringes are still visible if this distance is smaller than that between adjacent fringes, ≈*r*_o_*λ*/*d*. So, the approximate condition to see the fringes is *ξ* (*r*_o_/*ρ*_o_) *< r*_o_(*λ*/*d*), or:

d < ρ_o_λ/ξ. (13)

Equation (13) can be interpreted as follows: only radiation emitted within an area of diameter *ρ*_o_*λ*/*ξ* on the pinhole plane can contribute to diffraction, i.e., it is “laterally coherent”. 

By combining Equations (12) and (13), we discover an important upper limit for the parameter *η*:

*η* < [(*π*/4)(*ρ*_o_*λ*/*ξ*)^2^/*ρ*_o_^2^]/*Ω*, *η*_max_ = *πλ*^2^/(4*ξ*^2^*Ω*). (14)

The quantity *η*_max_ is called the “coherent power fraction” and measures how much “laterally coherent” radiation emits the source. In other words, it gauges the level of “lateral coherence”. 

Note that the coherent power fraction, and therefore the “lateral coherence”, increases if the source size *ξ* and/or the angular divergence *Ω* decrease. This illustrates the role of the source geometry in phase-contrast imaging. 

Practically speaking, the size *ξ* of a synchrotron radiation X-ray source corresponds to the cross-section of the electron beam circulating in the accelerator. This cross-section, and therefore *ξ*, are typically very small thanks to the sophisticated accelerator technology of today [3,4,9]. The vertical size now reaches the micron level, notably with advanced magnet “lattices” like the “multiple bend achromats”. 

Likewise, the angular divergence *Ω* of synchrotron sources is also very small. This is a phenomenon caused by Einstein’s relativity, which produces laser-like beams of X-rays [3,4,9].

In essence, synchrotron X-ray sources reach high levels of longitudinal and lateral coherence, fully suitable for phase-contrast radiography. This solved a big historical problem. Both the conditions for “longitudinal coherence” (Equations (7) and (11)) and for “lateral coherence” (Equation (14)) depend on the wavelength *λ*. And are much more difficult to satisfy for short X-ray wavelengths than for visible light. This explains why coherence-related applications started for X-rays only in the recent decades, whereas they have been present for centuries in visible optics.

**Figure 18:**
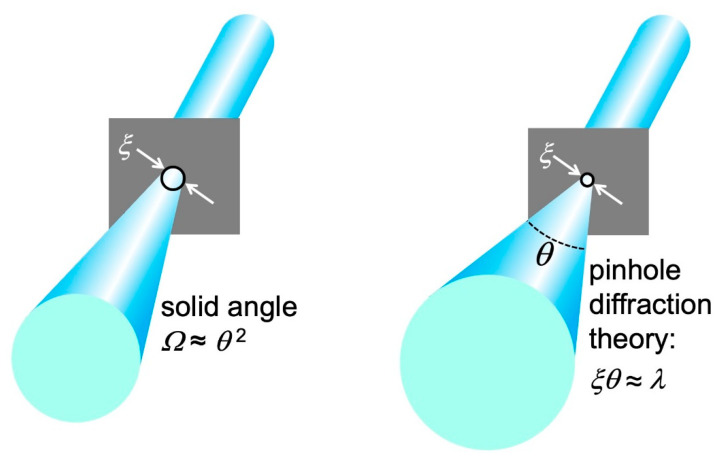
An attempt to obtain a small-area source using a shield with a pinhole reveals the diffraction limit for “lateral coherence”.

Before concluding our elementary discussion, we must discover a fundamental limit affecting “lateral coherence”. Consider a hypothetic procedure (Figure 18) to artificially obtain a small size source by partially blocking a beam using a shield with a pinhole. This produces a new “source”, the pinhole, with a size corresponding to its diameter *ξ*. 

However, pinhole diffraction increases the angular divergence of the radiation as *ξ* decreases. Elementary optics shows that the minimum value of the product *ξ*^2^*Ω* equals *λ*^2^. Thus, the coherent power fraction *η*_max_ defined by Equation (14) cannot exceed the value *π*/4.

This is a fundamental property of physics, called “diffraction limit”. The maximum value *π*/4 cannot be surpassed by improving the instrumentation. When a source reaches this limit, it has full “lateral coherence”. 

Many of the synchrotron sources of today reach the diffraction limit at least in part of their emitted spectrum of wavelengths. This is true, in particular, for the new class of X-ray sources called “free-electron lasers” [9]. 

## 4. Practical Use of X-ray Coherence 

Concrete applications of coherence-based radiography impact many different areas of science and technology. The examples are becoming very numerous: selecting a subset is not trivial. We shall concentrate here on some of our results in materials science, the life sciences, and cultural heritage, selecting examples that provide a good idea of the power and flexibility of the novel radiography techniques.

**Figure 19:**
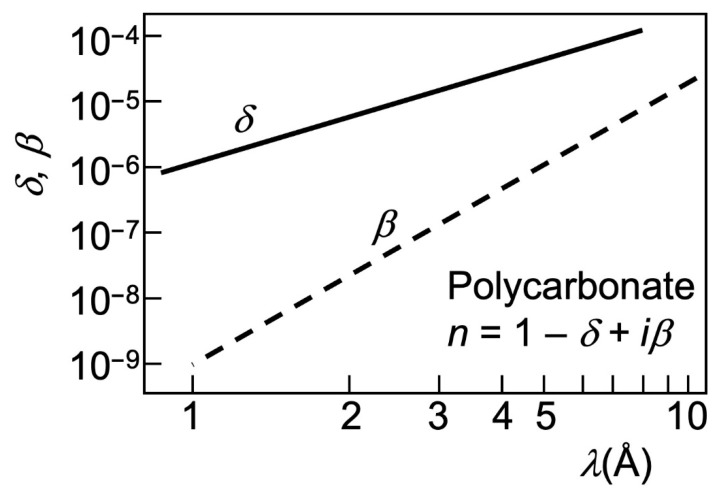
Wavelength dependence of the X-ray absorption coefficient *β* for polycarbonate, compared to the refractive index parameter *δ* (data from [17].

Before introducing such examples, we can anticipate that the dominating mechanism for all of them is once again refraction-induced edge enhancement. Absorption also contributes to the images, but its effects are weaker. This can be understood from Figure 19, which compares the parameters *δ* (defining the refractive index *n* with Equation (2)) and *β*, the absorption coefficient. 

Both parameters are small, so the corresponding effects—refraction, interference, and diffraction for *δ* and absorption for *β*—are limited. We see, however, that the effects of absorption are comparatively weaker. So, by not exploiting contrast mechanisms other than absorption one would miss excellent research opportunities, as clearly illustrated by the following examples.

### 4.1. Building on Bubbles

Edge-enhanced radiographs taken with non-monochromatized synchrotron radiation reach excellent signal-to-noise ratios and contrast levels. This can be exploited to detect minute object details that are practically invisible in conventional absorption radiography. Furthermore, it opens the door to dynamic experiments, which image-evolving systems on short time scales.

Tsai, W. L. et. al. (1996) reports a relevant example of such time-evolution studies [27]. The investigated process was metal electrodeposition. This is not a recent technique, but an old and commonly used one. Paradoxically, however, some of its aspects are still unclear, impacting the quality of its products.

It is known, for example, that in some cases electrodeposition of metal coatings yields poor results: visually unattractive rough-looking surfaces. A mechanism was proposed long ago to explain these failures and seek a remedy. But the hypothesized phenomenon was difficult for many to be accepted as plausible—until coherent X-ray radiography demonstrated its existence with real-time movies.

During electrodeposition, gas bubbles are formed close to the surface that is coated. The hypothesis was that the metal overlayer, under certain conditions, does not initially form on the substrate itself but on the surfaces of the bubbles. These then dynamically disappear, leaving cavities at the substrate-coating interface. Such leftover cavities can cause the poor quality of the coating.

**Figure 20:**
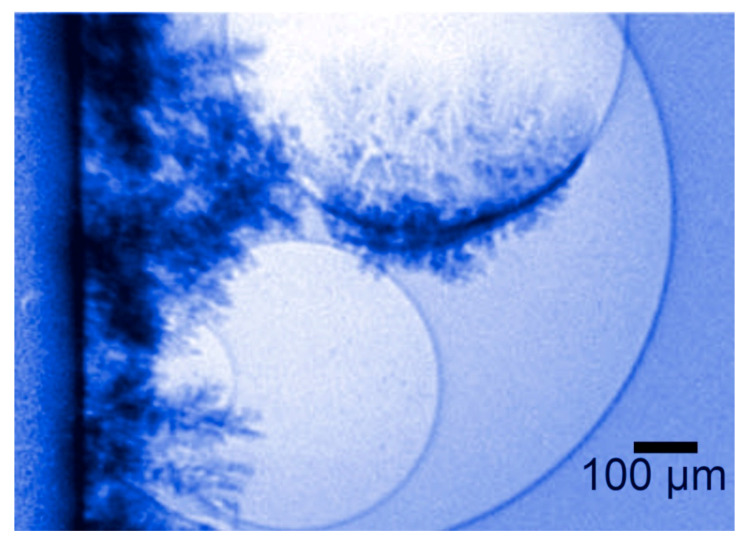
Coherence-based radiography proves a phenomenon that was previously difficult to believe: the growth of metal layers on the surface of gas bubbles during an electrodeposition process [27].

Experimental tests with phase-contrast movies demonstrated the validity of this hypothesis in a very direct way. Edge enhancement by refraction is indeed ideal to visualize bubbles. And metal coatings strongly absorb X-rays, becoming very visible. The combination of these features allowed taking real-time movies of the process, as shown in Figure 20.

The results were unmistakable and illustrated the power of this type of X-ray imaging. The hypothesized phenomenon was clearly visible and could be analyzed in detail, in view of a possible optimization of the deposition parameters to avoid defects. 

No matter how hard to believe, the “building on bubbles” mechanism could no longer be doubted after being directly seen [27]. No data processing at all was needed in this case to clarify a long-standing technological issue. 

### 4.2. Radiography of Microscopic Vessels: How Fireflies Luminesce

Vessels in living organisms are particularly difficult to image with conventional absorption-contrast radiography. In fact, the contrast is particularly weak between different organic tissues. This has very important consequences. For example, radiographic screening of heart diseases related to coronary arteries is still quite difficult, invasive, and not widely used.

The negative consequences of limited contrast also impact life science research. Indeed, it makes it difficult to detect small vessels, which often play a fundamental role in physiological mechanisms.

Tsai, Y. L. et al. (2002) presents a particularly challenging example of this problem [28]. The explored phenomenon was the emission of light by fireflies. This is both a fascinating mechanism and a very intriguing one.

The emitted power is indeed astonishing. If scaled up to a flying plane, the emission would likely cause it to crash. Thus, nature implements in fireflies an exceedingly effective light-producing process that could inspire human-oriented technologies.

**Figure 21:**
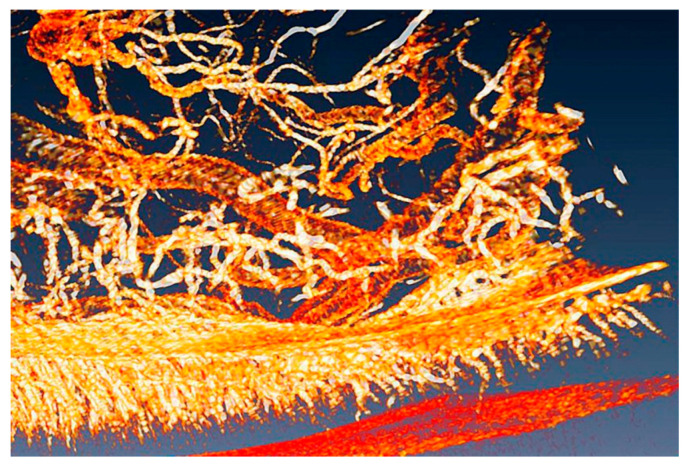
The complicated organ that provides the gas flow for the luminescence of fireflies. Phase-contrast radiography revealed even the smallest tubes, allowing reliable quantitative estimates [28]. Vertical image size ≈ 4 mm.

The phenomenon is not yet entirely explained and different hypotheses have been proposed for it. The emission takes place by breaking the compound (4*S*)-2-(6-hydroxy-1,3-benzothiazol-2-yl)-4,5-dihydrothiazole-4-carboxylic acid, also known as “firefly luciferin”. This requires supplying oxygen, but until recently it was not yet fully clear how this is accomplished in the firefly organ “lantern”. Part of this organ is visualized in Figure 21 using “coherent tomography”, a variant of phase-contrast imaging that will be discussed later.

One can see in the picture a very complicated system of microtubes that carry the oxygen to the site where it is needed for breaking luciferin. Some of the vessels are very small and not detectable with conventional absorption radiography.

This complicates the quantitative assessments of the oxygen-supplying process. Which, however, are necessary to identify the real mechanism among those that have been proposed. 

As seen in Figure 21, phase contrast detects instead even the smallest vessels, presenting a complete picture and allowing reliable quantitative assessments of the oxygen distribution process. This led to a fundamental discovery: to emit light, the insect diverts oxygen from other biological functions in order to make it available for breaking luciferin [28]. In particular, cellular energy production decreases temporarily.

Important details of firefly luminescence remain to be clarified. But phase-contrast radiography solved one big open question, validating a specific subset of models. 

The interest in this result goes beyond fundamental biology. If emulated, the mechanism could very effectively solve interesting technological problems, and lead to widespread applications. 

### 4.3. Phase-Contrast Radiography for Cultural Heritage Research

The novel coherence-based radiography techniques find interesting applications in cultural heritage studies. One intriguing example is the analysis of ancient handwritings [29,30,31]. This task is justified by the huge number of manuscripts that are present in European collections but are mostly useless for research.

In fact, the sheer mass of information is a deterrent for scholars. To be useful for them, the specimens should be digitized in a form suitable for automated data mining, processing, and tracing. The first step is of course systematic image taking.

Massive efforts are underway in this direction, but the task is gigantic. As an example, just the “Archivio di Stato” of Venice would take many decades to be fully digitalized. And there exist many comparably large collections throughout Europe, with huge amounts of buried information. Whose analysis could, for example, inject new realism into our view of history.

X-ray imaging could provide a suitable alternative to conventional picture-taking with visible light, offering at least two advantages. First, radiographic images of books can be obtained without opening them, minimizing the manipulation and the consequent risk of damage while increasing the image-taking speed. Second, advanced radiographic techniques can handle not only normal books but also problematic specimens like scrolls.

These advantages notwithstanding, one basic question had to be solved before launching the radiological analysis of handwritings: can the inks produce enough X-ray contrast? Extensive tests [29,30,31] gave a positive answer. In essence, the writing techniques in Europe remained almost invariant for a millennium. Nearly all the handwritings used variations of a basic recipe: the “iron gall” ink [29,30,31]. As the name suggests, this ink contains heavy metal iron which can produce strong absorption contrast for X-rays.

**Figure 22:**
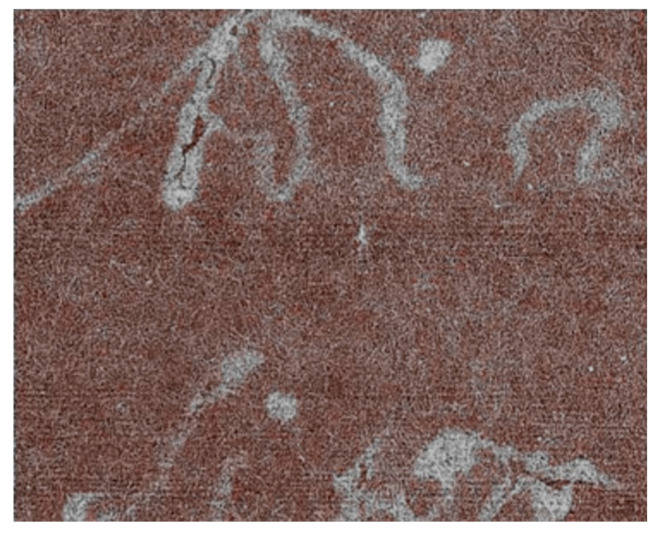
Radiography reads ancient handwritings with “iron gall” inks without requiring the opening of books [29,30,31].

**Figure 23:**
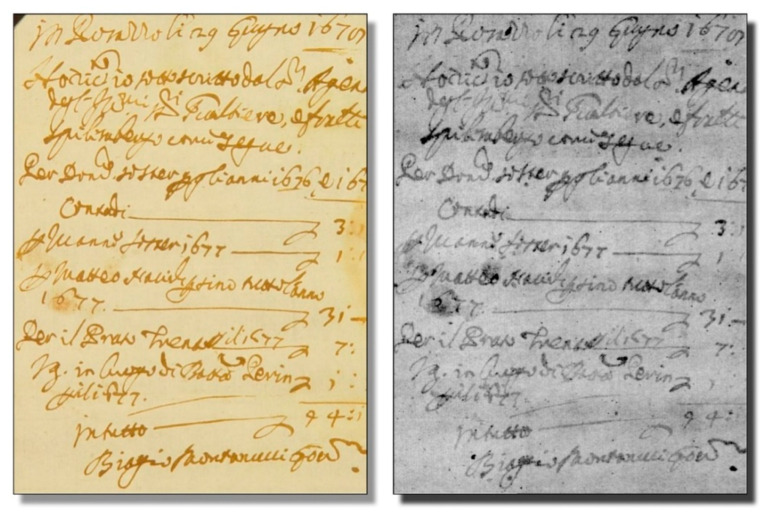
A manuscript revealed by radiography (right), compared to the corresponding visible-light picture [29,30,31].

Figure 22 and Figure 23 show examples of the results of this approach, which was developed under the leadership of Fauzia Albertin [29,30,31]. Different techniques have been successfully applied to this task, including phase contrast and tomographic reconstruction. And the analysis of thick books has already been successfully tested.

## 5. Advanced Coherence-Based X-ray Imaging Techniques

Our discussion was so far focused on one kind of phase-contrast mechanism, refraction edge enhancement. We already mentioned, however, that other wave-like phenomena based on coherence can be exploited for radiography. They led to a number of specialized techniques with many potential applications. Our scope here is not to present an exhaustive picture, but some examples that could stimulate the readers’ appetite for more information and perhaps their practical ideas for utilizations. For a more comprehensive discussion of coherence-based radiography techniques, the reader can consult Refs. [32,33,34,35,36,37] and the references therein.

### 5.1. Phase-Contrast Tomography 

The first example is the coherence-based counterpart of one of the most popular absorption radiography techniques: computer-assisted tomography (CAT). As it is well known, CAT produces three-dimensional radiographic pictures of objects with excellent flexibility in selecting the viewing mode. Thus, it has become a very widely used instrument for medical diagnostics.

The foundation of CAT is the three-dimensional information present in a set of two-dimensional (“projection”) radiographic images taken from different viewpoints. A typical CAT set consists of several thousand “projection” pictures acquired in different directions, by rotating the radiography apparatus or, alternatively, the object. Pictures are normally taken at regular angular intervals.

From a CAT set, well-established computer algorithms can reconstruct virtual images of different object parts, seen from different points of view and with adjustable depth penetrations. Over the past several decades, absorption-based CAT has become a very sophisticated and very powerful instrument.

The advent of phase contrast in X-ray imaging triggered the obvious question: could it be implemented in the CAT mode? The issue was not trivial, since the information yielded by a “phase object” is not equivalent to that from an “absorption object”. And the CAT reconstruction techniques had been specifically developed for absorption images. Could they be applied to phase-contrast images, and if yes to what extent?

**Figure 24:**
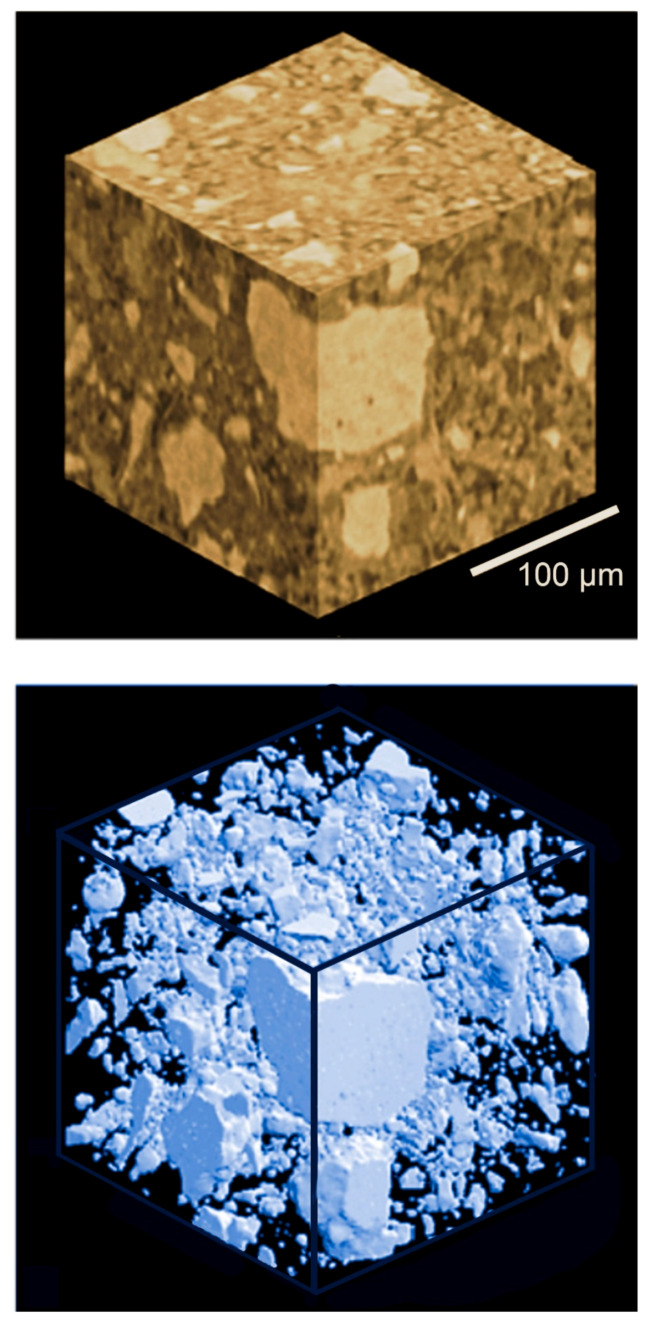
Example of coherence-based tomography: two reconstructions of a sample of concrete [39].

**Figure 25:**
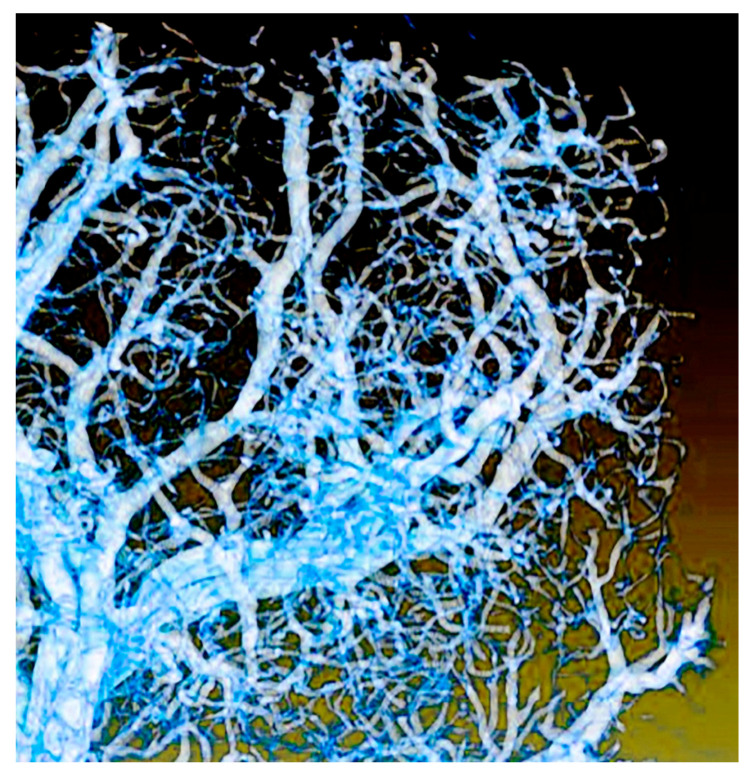
Another example of coherence-based tomography: microvasculature developed in the early stages of tumor growth [38]. Image size 600 µm.

Once again, practical tests provided the answer. Phase-contrast tomography was successfully implemented in many cases, delivering useful and rather spectacular results [14,28,38,39]. Figure 24 and Figure 25 show two examples of this very successful approach [32,33]. Also note that some of the results previously introduced in this text were obtained with tomography, specifically those of Figure 5 and Figure 20.

Phase-contrast tomography has become by now a routine technique. Its main practical problem is the need to take large sets of projection images, which increases the duration of the experiment. As we shall see in the last section of this text, excellent progress has been recently made to tackle this issue.

### 5.2. Diffraction-Enhanced Imaging

Our previous discussion, mostly limited to X-ray refraction and/or absorption, was somewhat oversimplified. In fact, several other phenomena contribute to X-ray image formation. The corresponding, valuable information can be extracted with experimental strategies different from those described so far.

For example, monochromatized synchrotron radiation can analyze phenomena occurring at specific wavelengths. And sophisticated techniques for spatial and/or spectral filtering of the X-rays can separate the different contributions to the images.

One very relevant example is “Diffraction Enhanced Imaging” (DEI) [12,40,41]. Which achieves the separation of different contributions to the images using monochromatized synchrotron radiation and a filtering crystal to process the X-rays after they interact with the object. The inventors of DEI demonstrated [40] that with clever use of the experimental geometry and minimal data processing one can obtain separated “absorption” and “diffraction” images.

**Figure 26:**
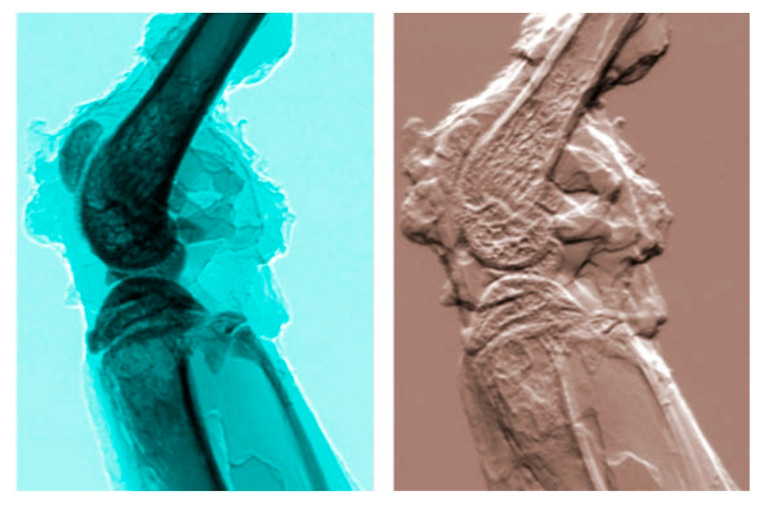
Example of DEI (diffraction-enhanced imaging) [40]: a mouse leg imaged with absorption radiography (left) and with DEI (refractive index) (see [41], picture reproduced by permission).

Figure 26 shows a nice example of DEI results [41]. The image quality is objectively spectacular. The absence of background intensity is particularly attractive, especially for potential medical diagnostics applications such as mammography.

## 6. Towards the Future: The SYNAPSE Project

What will be the long-term impact of coherence-based radiography? Difficult to predict, for a number of reasons. But to have any impact at all, the new techniques must be massively applied to real problems in science and technology. 

Unfortunately, there exists in this domain a certain tendency to favor the development of experimental techniques over their practical applications. This is not good: unused techniques, no matter how clever and sophisticated, are of no value. And it is not easy to transform new techniques from mere feasibility tests to practical tools.

This transformation is further complicated if the objective is medical diagnostics. In fact, such an objective is affected by many other technical and non-technical issues. The efforts towards broader practical applications are easier—and desirable—for materials science and biology. 

**Figure 27:**
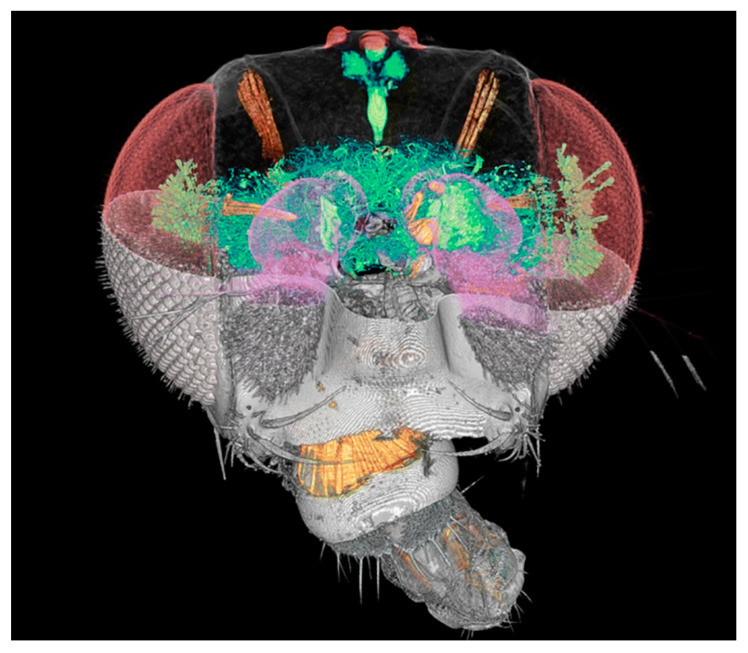
Coherence-based tomographic reconstruction of a drosophila fly head [36,37].

We shall illustrate these points with a specific and very relevant project: SYNAPSE, whose objective is to map large animal and human brains. Its ambition is to obtain comprehensive charts of all neuron cells and of their connections. One of the key achievements prior to SYNAPSE [42,43] was mapping the Drosophila fly brain (Figure 27). The ultimate dream is, of course, to map the “connectome” of the entire human brain.

The development of practical strategies to transform such a dream into reality reveals the formidable obstacles that separate feasibility tests from real-life experiments. These are the most important.

Spatial resolution: the radiography techniques must be able to detect details of the objects on a scale suitable for brain mapping. One must consider two resolution levels. At the micron scale, the edge-enhancement techniques previously described are adequate and yield good general images of brain cells with some details. However, regions of special interest must be imaged with a better, nanometer-scale resolution. 

This is achieved with the microscopy techniques discussed in Section 2.5. However, using nanoscale resolution increases the image-taking time and the duration of the experiments. This is a key problem, as discussed in the next point.

Total duration of the experimental program: mapping the Drosophila brain required a few years [42,43]. Most of this time was spent taking projection images for tomography sets, whereas image processing had a rather marginal impact. Scaled up to a human brain, the duration of SYNAPSE would become unmanageable. Hence, image taking must be drastically accelerated. This was already partially achieved, but further progress is necessary. And it is necessary [10] to engage in the program several synchrotron facilities in different countries, operating in parallel in a coordinated fashion. This is the basic philosophy of SYNAPSE. 

Image contrast: microscopic soft tissue features in brain specimens such as organelles, axons, and synapses do not offer much contrast for X-ray imaging, even in the presence of edge enhancement. So, the contrast must be boosted by staining the objects [42,43]. The common procedure for brain specimens is Golgi staining. Coherence is nevertheless critical in achieving high resolution with sufficient contrast on these submicron metal-stained features [22]. 

Radiation damage: this is a potential issue for all radiological studies at the cellular level. The problem is typically handled with empirical approaches, systematically checking the specimens for possible modifications. So far, the results have been negative, but systematic assessments are required.

Data processing: the project of human brain mapping is primarily based on tomography and requires computer reconstructions of very many large tomographic image sets. The amount of data to be processed for SYNAPSE is enormous: it would largely exceed the present resources of organizations like Google. Once again, a coordinated effort over different countries appears necessary [10]. 

Data storage, availability, and security: the objective of SYNAPSE is to make its data available to all qualified researchers worldwide. But the sheer dimension of the information sets creates extreme problems. Access should be guaranteed without barriers and across national borders. But this creates formidable problems of data security. Once again, there is a big gap between feasibility tests and real experiments that handle real data.

How can all these challenges be managed? SYNAPSE [10] is a concrete response: a recently launched multinational program, based on a coalition of synchrotron facilities operating in different countries within the Asia-Pacific basin. 

At this time, SYNAPSE involves Japan, South Korea, China, Australia, Taiwan, and Singapore, with foreseen extensions to other countries. Its present partners are PAL (Pohang Accelerator Laboratory, Pohang, Korea) RIKEN/Spring-8, Shanghai/IoN (Institute of Neuroscience, Shanghai, China), SSLS/NUS (Singapore Synchrotron Light Source, National University of Singapore, Singapore), Yong Loo Lin School of Medicine (NUS), the Institute of Physics of the Academia Sinica (Taipei, Taiwan), NSSRC (National Synchrotron Radiation Research Center, Hsinchu, Taiwan) and the Australian Synchrotron (Clayton, Australia, ANSTO).

SYNAPSE is built on the success of the previous program AXON (Accelerated X-ray Observation of Neurons). Which produced, in particular, important technical results as far as accelerating the image taking is concerned [10,42,43]. 

The SYNAPSE coalition was formally inaugurated early in 2020 with a ceremony in Singapore. In spite of the unforeseen problems caused by the Covid-19 pandemics, the programs initiated and already produced good results [10]. The objective to map one human brain within the next few years appears feasible.

SYNAPSE is also tackling the huge problems of data processing, storage, security, and availability. This is handled by a consortium of high-performance computing institutions in the participating countries, under the leadership of the National Supercomputer Center in Singapore [10]. 

**Figure 28:**
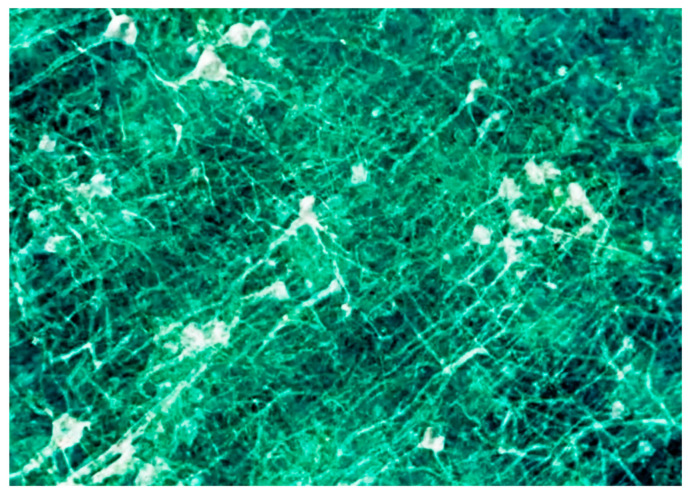
Neuron network specimen, one of the first coherence-based radiography results obtained under the SYNAPSE program [10].

**Figure 29:**
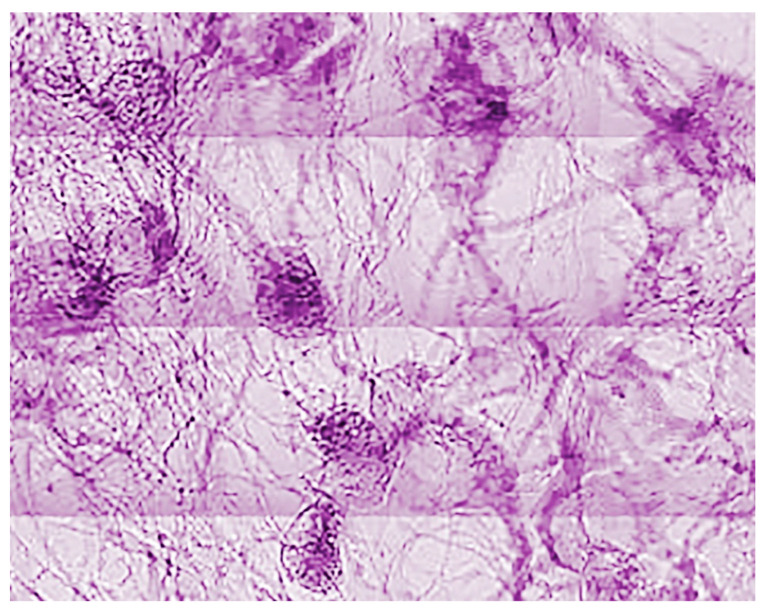
Another early result of SYNAPSE [10].

Figure 28 and Figure 29 show some of the first results produced under the SYNAPSE umbrella [10]. Note that this gigantic effort is not only important for the fundamental understanding of physiological functions, but also to study and handle brain diseases with enormous social consequences. Furthermore, the X-ray imaging and computing technologies developed under SYNAPSE will impact a variety of science and technology fields, with the potential for widespread applications.

## Data Availability

Not applicable.

## Appendix A

This is the derivation of Equation (3) based on the geometry of Figure 8, using Equation (2) for the refractive index *n*, with *δ* << 1.

One can see in the bottom-right part of Figure 8 that the incidence angle of the X-ray beam on the left-hand edge surface equals the edge slanting angle *α*. Refraction at this surface changes the initial beam direction by an angle *θ* according to the Snell law:

sin*θ*/sin*α* = 1/*n* = 1/(1 − *δ*) ≈ 1 + *δ* (A1)

The deviation angle *μ*_1_ for the left-hand edge surface equals *θ* − *α*, thus:

sin*μ*_1_ = sin(*θ* − *α*) = sin*θ* cos*α* − sin*α* cos*θ* ≈ (1 + *δ*)sin*α*cos*α* − sin*α* (1 − sin^2^*θ*)^1/2^ ≈ (1 + *δ*)sin*α*cos*α* − sin*α* [1 − (1 + *δ*)^2^sin^2^*α*]^1/2^ ≈ (1 + *δ*)sin*α*cos*α* − sin*α* [1 − (1 + 2*δ*)sin^2^*α*]^1/2^ = (1 + *δ*)sin*α*cos*α* − sin*α* (cos^2^*α* − 2*δ*sin^2^*α*)^1/2^ = sin*α*cos*α* [(1 + *δ*) − (1 − 2*δ* tg^2^*α*)^1/2^] ≈ sin*α*cos*α* [(1 + *δ*) − (1 + *δ* tg^2^*α*)] = sin*α*cos*α* (1 + tg^2^*α*)*δ* = sin*α*cos*α* (1/cos^2^*α*)*δ* = (tg*α*)*δ*. (A2)

We must now connect the deviation angles *μ*_1_ and *μ* using Snell’s law for the right-hand edge surface:

sin*μ* ≈ *n*sin *μ*_1_ ≈ sin *μ*_1_ ≈ (tg*α*)*δ*, (A3)

which for small deviation angles *μ* demonstrates Equation (3).

## References

[1] Röntgen W.C. (1895). Über eine neue Art von Strahlen. Sitzungsberichte der Physikalisch.

[2] Thomas A.M.J., Banerjee A. (2013). The History of Radiology.

[3] Margaritondo G. (2002). Elements of Synchrotron Light for Biology, Chemistry, and Medical Research.

[4] Margaritondo G. (1988). Introduction to Synchrotron Radiation.

[5] Hwu Y., Tsai W.L., Groso A., Margaritondo G., Je J.-H. (2002). Coherence-enhanced Synchrotron Radiology: Simple Theory and Practical Applications. J. Phys..

[6] Stampanoni M., Menzel A., Watts B., Mader K.S., Bunk O. (2014). Coherent X-ray Imaging: Bridging the Gap Between Atomic and Micro-scale Investigations. Chimia.

[7] Munro P.R.T. (2019). Coherent X-ray Imaging Across Length Scales. J. Contemp. Phys..

[8] Margaritondo G., Di Cicco A., Giuli G., Trapananti A. (2021). Coherence: Elementary Introduction to a Quantum Revolution in X-Ray Science. Synchrotron Radiation Science and Applications.

[9] Margaritondo G., Hwu Y. (2021). Synchrotron Radiation and X-ray Free Electron Lasers (X-FELs) Explained to all Users, Active and Potential. J. Synchrotron Radiat..

[10] SYNAPSE https://synapse-sg.org/.

[11] Hwu Y., Tsai W.L., Chang H.M., Yeh H.I., Hsu P.C., Yang Y.C., Su Y.T., Tsai H.L., Chow G.M., Ho P.C. (2004). Imaging Cells and Tissues with Refractive Index Radiology. Biophys. J..

[12] Meuli R., Hwu Y., Je J.H., Margaritondo G. (2004). Synchrotron Radiation in Radiology—Part II: Radiology Techniques Based on Synchrotron Sources. Eur. Radiol..

[13] Margaritondo G., Hwu Y., Je J.H. (2004). Synchrotron Light in Medical and Materials Science Radiology. Rivista Nuovo Cimento.

[14] Margaritondo G., Hwu Y., Je J.H. (2008). Nondestructive Characterization by Advanced Synchrotron Light Techniques: Spectromicroscopy and Coherent Radiology. Sensors.

[15] Margaritondo G., Tromba G. (1999). Coherence-Based Edge Diffraction Sharpening of X-Ray Images: A Simple Model. J. Appl. Phys..

[16] Hwu Y., Hsieh H.H., Lü M.J., Tsai W.L., Lin H.M., Goh W.C., Lai B., Je J.H., Kim C.K., Noh D.Y. (1999). Coherence-enhanced Synchrotron Radiology: Refraction Versus Diffraction Mechanisms. J. Appl. Phys..

[17] X-ray Parameters (Henke Tables). https://henke.lbl.gov/optical_constants/.

[18] Wu S.-R., Hwu Y., Margaritondo G. (2012). Hard-X-ray Zone Plates: Recent Progress. Materials.

[19] Chu Y.S., Yi J.M., De Carlo F., Shen Q., Lee W.-K., Wu H.J., Wang C.L., Wang J.Y., Liu C.J., Wang C.H. (2008). Hard-X-ray Microscopy with Fresnel Zone Plates Reaches 40 nm Rayleigh Resolution. Appl. Phys. Lett..

[20] Seol S.K., Kim J.T., Je J.H., Hwu Y., Margaritondo G. (2008). Three-Dimensional (3D) Polypyrrole Microstructures with High Aspect Ratios Fabricated by Localized Electropolymerization. Macromolecules.

[21] Chen Y.T., Lo T.N., Chu Y.S., Yi J., Liu C.J., Wang J.Y., Wang C.L., Chiu C.W., Hua T.E., Hwu Y. (2008). Full-field Hard X-Ray Microscopy below 30 Nanometers: A Challenging Nanofabrication Achievement. Nanotechnology.

[22] Chen Y.-T., Chen T.-Y., Yi J., Chu Y.S., Lee I., Wang C.-L., Kempson I., Hwu Y., Gajdosik V., Margaritondo G. (2011). Hard X-ray Zernike Microscopy Reaches 30 nm Resolution. Opt. Lett..

[23] Yi J., Chu Y., Chen Y., Chen T., Hwu Y., Margaritondo G. (2011). High-resolution Hard-X-ray Microscopy Using Second-order Zone Plate Diffraction. J. Phys. D.

[24] Wu S.R., Lin C.H., Chen Y.S., Hwu Y., Chu Y.S., Margaritondo G., Chen Y.Y. (2011). At the Frontiers of High-resolution Hard-x-ray Microscopy: An International Programme. J. Phys. D.

[25] Welnak J., Dong Z., Solak H., Wallace J., Cerrina F., Bertolo M., Bianco A., Di Fonzo S., Fontana S., Jark W. (1995). SUPERMAXIMUM: A Schwarzschild-based Spectromicroscope for ELETTRA. Rev. Sci. Instrum..

[26] Snigirev A., Kohn V., Snigireva I., Lengeler B. (1996). A Compound Refractive Lens for Focusing High-energy X-rays. Lett. Nat..

[27] Tsai W.L., Hsu P.C., Hwu Y., Chen C.H., Chang L., Je J.H., Lin H.M., Groso A., Margaritondo G. (2002). Building on Bubbles in Metal Electrodeposition. Nature.

[28] Tsai Y.-L., Li C.-W., Hong T.-M., Ho J.-Z., Yang E.-C., Wu W.-Y., Margaritondo G., Hsu S.-T., Ong E.B.L., Hwu Y. (2014). Firefly Light Flashing: Oxygen Supply Mechanism. Phys. Rev. Lett..

[29] Albertin F., Astolfo A., Stampanoni M., Peccenini E., Hwu Y., Kaplan F., Margaritondo G. (2015). X-ray Spectrometry and Imaging for Ancient Administrative Handwritten Documents. X-ray Spectrom..

[30] Albertin F., Astolfo A., Stampanoni M., Peccenini E., Hwu Y., Kaplan F., Margaritondo G. (2015). Ancient Administrative Handwritten Documents: Virtual X-ray Reading. J. Synchrotron Radiat..

[31] Albertin F., Patera A., Jerjen I., Hartmann S., Peccenini E., Kaplan F., Stampanoni M., Kaufmann R., Margaritondo G. (2016). Virtual Reading of a Large Ancient Handwritten Science Book. Microchem. J..

[32] Phase-Contrast X-Ray Imaging. https://en.wikipedia.org/wiki/Phase-contrast_x-ray_imaging.

[33] Momose A. (2002). Phase Contrast X-ray Imaging Based on Interferometry. J. Synchrotron Radiat..

[34] Tao S., He C., Hao X., Kuang C., Liu X. (2021). Principles of Different X-ray Contrast Imaging: A Review. Appl. Sci..

[35] Mayo S.C., Stevenson A.W., Wilkins S.W. (2012). In-line Phase Contrast X-ray Imaging and Tomography for Materials Science. J. Synchrotron Radiat..

[36] Stampanoni M., Mokso R., Marone F., Vila-Comamala J., Gorelick S., Trtik P., Jefimovs K., David C. (2010). Phase Contrast Tomography at the Nanoscale Using Hard X-rays. Phys. Rev..

[37] Howells M. Coherent X-rays: Overview. https://www.esrf.fr/files/live/sites/www/files/events/conferences/Tutorials/slideslecture1.pdf.

[38] Margaritondo G. (2017). Synchrotron Light: A Success Story over Six Decades. Rivista Nuovo Cimento.

[39] Gallucci E., Scrivener K., Groso A., Stampanoni M., Margaritondo G. (2007). 3D Experimental Investigation of the Microstructure of Cement Pastes Using Synchrotron X-ray Microtomography (µCT). Cement Concrete Res..

[40] Chapman D., Thomlinson W., Johnston E.R., Washburn D., Pisano E., Gmür N., Zhong Z., Menk R.H., Arfelli F., Sayers D. (1997). Diffraction Enhanced X-ray Imaging. Phys. Med. Biol..

[41] Menk R.H., Rigon L., Arfelli F. (2005). Diffraction-Enhanced X-ray Medical Imaging at the ELETTRA Synchrotron Light Source. Nucl. Instrum. Meth. Phys. Res..

[42] Hwu Y., Margaritondo G., Chiang A.-S. (2017). Why Use Synchrotron X-ray Tomography for Multi-scale Connectome Mapping?. BMC Biol..

[43] Chin A.-L., Yang S.-M., Chen H.-H., Li M.-T., Lee T.-T., Chen Y.-J., Lee T.-K., Petibois C., Cai X., Low C.-M. (2020). A Synchrotron X-ray Imaging Strategy to Map Large Animal Brains. Chin. J. Phys..

